# Psoriasis Carries an Increased Risk of Venous Thromboembolism: A Danish Nationwide Cohort Study

**DOI:** 10.1371/journal.pone.0018125

**Published:** 2011-03-25

**Authors:** Ole Ahlehoff, Gunnar Hilmar Gislason, Jesper Lindhardsen, Mette Gitz Charlot, Casper Haslund Jørgensen, Jonas Bjerring Olesen, Ditte-Marie Bretler, Lone Skov, Christian Torp-Pedersen, Peter Riis Hansen

**Affiliations:** 1 Department of Cardiology, Copenhagen University Hospital Gentofte, Hellerup, Denmark; 2 Department of Dermatology, Copenhagen University Hospital Gentofte, Hellerup, Denmark; 3 Faculty of Health Sciences, University of Copenhagen, Copenhagen, Denmark; Innsbruck Medical University, Austria

## Abstract

**Background:**

Psoriasis is an immunoinflammatory disease associated with cardiovascular risk factors, atherothrombotic events, and hypercoagulability. Venous thromboembolism (VTE) is potentially lethal and shares risk factors with psoriasis, but the risk of VTE associated with psoriasis is unknown. The present study investigated the potential association between psoriasis and VTE.

**Methods and Findings:**

Information from nationwide prospectively recorded registers of hospitalization, drug dispensing from pharmacies, socio-economic data, and causes of death was linked on an individual level. In an unselected nationwide cohort, we used multivariate Poisson regression models controlling for age, gender, comorbidity, concomitant medication, socio-economic data, and calendar year, to assess the risk of VTE associated with psoriasis. A total of 35,138 patients with mild and 3,526 patients with severe psoriasis were identified and compared with 4,126,075 controls. Patients with psoriasis had higher incidence rates per 1000 person-years of VTE than controls (1.29, 1.92, and 3.20 for controls, mild psoriasis, and severe psoriasis, respectively). The rate ratio (RR) of VTE was elevated in all patients with psoriasis with RR 1.35 (95% confidence interval [CI] 1.21–1.49) and RR 2.06 (CI 1.63–2.61) for mild and severe psoriasis, respectively. Exclusion of patients with malignancies, and censoring of patients undergoing surgery did not alter the results.

**Conclusion:**

This nationwide cohort study indicates that patients with psoriasis are at increased risk of VTE. The risk was highest in young patients with severe psoriasis. Physicians should be aware that patients with psoriasis may be at increased risk of both venous and arterial thromboembolic events.

## Introduction

Psoriasis is a prevalent chronic immunoinflammatory disease affecting approximately 2% of the population [1], [2]. It is associated with cardiovascular risk factors, atherothrombotic events, and markers of hypercoagulability, including platelet activation and hyperhomocysteinemia [3]–[15]. Venous thromboembolism (VTE), i.e., deep venous thrombosis and pulmonary embolism, is prevalent and potentially lethal, and is associated with various conditions including cancer, prolonged immobilization, and major surgery or trauma (secondary VTE) [16]. Interestingly, VTE is also associated with certain cardiovascular risk factors (e.g., obesity, hypertension, and smoking), and arterial cardiovascular events [16]–[18]. Moreover, the risk of VTE is associated with elevated levels of C-reactive protein [19], and increased risk of VTE was recently demonstrated in patients with inflammatory bowel disease [20], [21]. The potential impact of psoriasis on the risk of VTE, however, has not been examined in detail previously [6]. We therefore examined the risk of VTE in patients with psoriasis in a cohort study based on nationwide prospectively recorded registers with the underlying hypothesis that patients with psoriasis carry an increased risk of VTE.

## Methods

### Ethics

This study was approved by The Danish Data Protection Agency (2007-41-1667), and data at the individual case level were made available to us by the national registers in anonymized form. Register studies do not require ethical approval in Denmark. The authors had full access to all data and take full responsibility for its integrity.

### Study population and data sources

The study population comprised the entire Danish population aged ≥18 years on January 1, 1997. The population was followed until December 31, 2006, or death. Patients with prevalent psoriasis, a history of previous VTE, and patients receiving vitamin K antagonist treatment at baseline were not included. The study was conducted and reported in accordance with the Strengthening the Reporting of Observational Studies in Epidemiology (STROBE) recommendations [22]. In Denmark, all citizens have a unique personal civil registration number that enables individual level-linkage of information across nationwide prospectively recorded registers. All medications dispensed from pharmacies were obtained from the National Prescription Registry (the Danish Registry of Medicinal Product Statistics), where all dispensed prescriptions have been recorded since 1995 ensuring complete registration. Patients with psoriasis were identified by their claims of prescriptions for vitamin D derivatives. Patients were included when claiming their second prescription for these agents to ensure persistent medical treatment for psoriasis as previously accepted [12]. Subjects with prevalent psoriasis were defined as patients fulfilling the psoriasis criteria prior to study start. Morbidity was obtained from the Danish National Patient Register in which all hospital contacts, diagnoses, and invasive procedures have been recorded since 1978 according to the International Classification of Diseases (ICD), i.e. ICD-8 until 1994 and ICD-10 thereafter. Patients with severe psoriasis were identified by hospitalizations (including out-patient visits) for psoriasis (ICD-10 L40) or psoriatic arthritis (M070–M073) and included at the time of their third diagnosis. The severe psoriasis classification has previously been validated and pose an acceptable measure of severe disease [12]. Comorbidity at study entry was described by Charlson's index, as defined by 19 prespecified diagnoses at study entry and up to 1 year previously [23]. All deaths were identified from the Central Population Register, in which deaths are recorded within 2 weeks. Causes of death, recorded according to ICD codes, were obtained from the National Causes of Death Register. Socioeconomic status was defined by the individual average yearly gross income in a 5-year period prior to inclusion and patients were divided into quintiles according to their income.

### Medical treatment

Prescriptions claimed for topical vitamin-D derivatives (ATC D05AX), i.e., topical treatment used exclusively for psoriasis [2], and vitamin K antagonists (B01AA) were used for in- and exclusion of subjects as described above. Pharmacologically managed cardiovascular diseases and cardiovascular risk factors, including hypertension, dyslipidemia, and diabetes mellitus were identified by prescriptions for platelet inhibitors (B01AC), betablockers (C07), angiotensin-converting enzyme inhibitors (ACEI)/angiotensin 2 receptor antagonists (C09), loop diuretics (C03C), spironolactone (C03D), statins (C10A), and glucose-lowering drugs (A10) claimed up to 6 months prior to study initiation (Table 1).

**Table 1:** Baseline characteristics of the study population.

Characteristic	Controlsn = 4,126,075	Mild psoriasisn = 35,138	Severe psoriasisn = 3526
**Age, years (SD)**	46.8 (18)	47.7 (16)	48.4 (16)
**Men (%)**	2,016,289 (48.9)	17,554 (50.0)	1829(51.9)
**Women (%)**	2,109,786 (51.1)	17,584 (50.0)	1697(48.1)
**No. of person-years**	38,503,056	175,384	22,135
**Comorbidity (%)**			
Peripheral vascular disease	5609 (0.14)	43 (0.12)	8 (0.23)
Cerebrovascular disease	12,323 (0.30)	90 (0.26)	8 (0.23)
Coronary heart disease	19,453 (0.47)	190 (0.54)	37 (1.05)
Congestive heart failure	7327 (0.16)	41 (0.11)	9 (0.32)
Hepatic disease	2532 (0.06)	22 (0.06)	31 (0.88)
Chronic obstructive pulmonary disease	11,149 (0.27)	56 (0.16)	10 (0.28)
Cardiac dysrhythmia	11,115 (0.27)	66 (0.19)	16 (0.45)
Renal disease	2330 (0.06)	10 (0.03)	5 (0.14)
Cancer	24,856 (0.60)	156 (0.44)	35 (0.99)
Rheumatological disease	3746 (0.09)	28 (0.08)	9 (0.26)
**Treatment (%)**			
Platelet inhibitor	95,900 (2.32)	844 (2.40)	71 (2.01)
Beta-blocker	134,809 (3.27)	1499 (4.27)	167 (4.74)
ACEI/ARB	116,412 (2,82)	1244(3.54)	133 (3.77)
Loop diuretic	122,929 (2.98)	860(2.45)	151 (4.28)
Statin	27,950 (0.68)	371 (1.06)	33 (0.94)
Spironolactone	14,367 (0.35)	101(0.29)	11 (0.77)
Glucose-lowering drug	71,659 (1.74)	643 (1.83)	96 (2.72)

SD: standard deviation; ACEI: angiotensin-converting enzyme inhibitors; ARB: angiotensin II receptor blocker.

### Study endpoints

The following primary endpoint was assessed: first time in-hospital discharge diagnosis of VTE (ICD-10 I26 and I80.1–I80.9). VTE diagnoses made in emergency departments were not included. The diagnosis of VTE (deep venous thrombosis and pulmonary embolism) in hospitalized patients has previously been validated in the Danish National Patient Register with a positive predictive value of 75% when excluding diagnoses from emergency departments [24]. Hospitalizations with the specific diagnosis of pulmonary embolism (ICD-10 I26) were evaluated as a secondary endpoint.

### Statistical analysis

Baseline characteristic are presented as percentages and means with standard deviations. Unadjusted event rates are summarized as events per 1000 person-years. The rate ratios (RRs) and 95% confidence interval (CI) of VTE were estimated by time-dependent Poisson regression models adjusted for age, calendar year, concomitant medication, comorbidity, socioeconomic data, and gender. Psoriasis status was included as a time-dependent variable, i.e., patients were only considered at risk from the time they complied with the inclusion criteria. Age and calendar year were also included as time-dependent variables. Comorbidity, socioeconomics, and concomitant medication were included as fixed variables obtained at baseline.

Sensitivity analyses with exclusion of patients at increased risk of secondary VTE at baseline, including those with cancer (ICD-10 C00–C97), the risk of which may be increased in psoriatic patients [25], and rheumatological disease including psoriatic arthritis (ICD-10 M05–07.3, M32–34, and M35.3) were conducted. The potential impact of immobilization and hemostatic activation due to surgery (any surgical procedure code) was assessed in a sensitivity analysis with censoring of subjects at the time of the surgical procedure. Surveillance bias was addressed in analyses with inclusion of patients with psoriasis at the time of their first vitamin D prescription claim or first psoriasis diagnosis, and by exclusion of all subjects with a history of hospitalizations up to 1 year prior to study start. A two-sided p-value<0.05 was considered statistically significant. Interaction between severe psoriasis and age was found, and therefore both overall and age-stratified estimates are presented. All statistical analyses were performed with the use of SAS statistical software version 9.2 and STATA software version 11.

## Results

**Figure 1:**
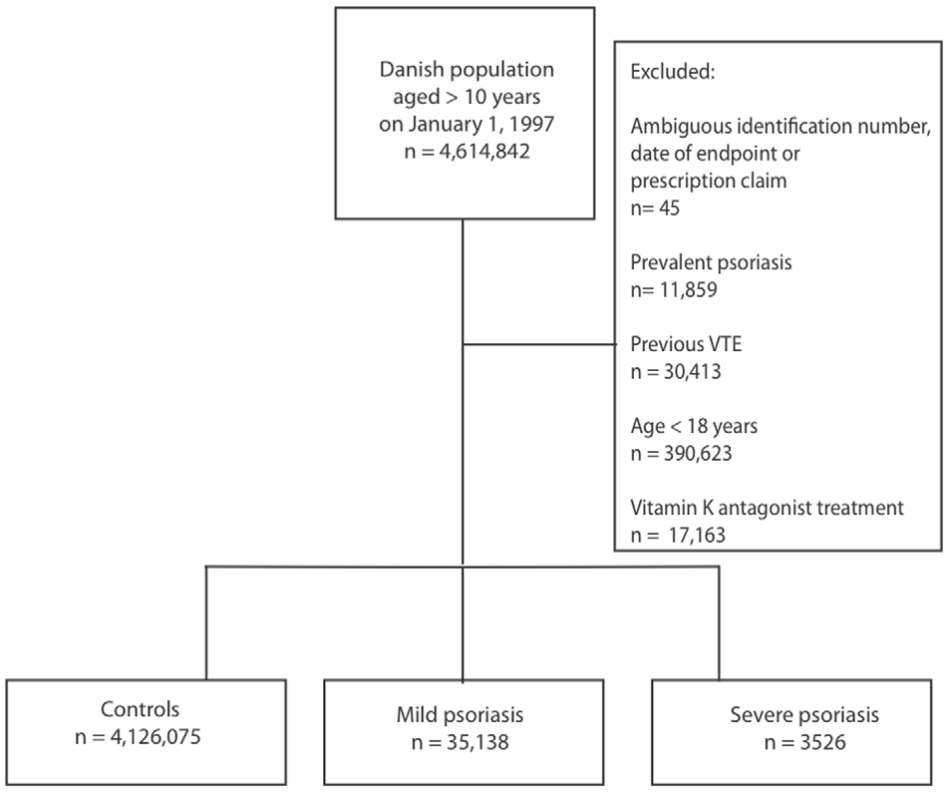
Flowchart of study population selection. VTE: venous thromboembolism.

The cohort study had a maximum follow-up of 10 years. A total of 35,138 patients with mild psoriasis, 3526 patients with severe psoriasis, and 4,126,075 controls were identified. Details on study population selection are presented in Figure 1. Patients with severe psoriasis were more often men and more often treated with cardiovascular medication and glucose-lowering drugs (Table 1). We have previously demonstrated an age- and severity-dependent increase in risk of cardiovascular and all-cause mortality with psoriasis in this nationwide cohort [12]. Patients with psoriasis had higher overall VTE rates than controls, i.e., 1.29, 1.92, and 3.20 per 1000 person-years for controls, mild psoriasis, and severe psoriasis, respectively. This pattern of increased VTE rates was sustained in the age-stratified event rates (Table 2), and in analyses of the secondary endpoint of pulmonary embolism, with corresponding overall pulmonary embolism rates 0.44, 0.61, and 1.02 per 1000 person-years.

**Table 2:** Age-stratified incidence rates per 1000 person-years.

	Controls	Mild psoriasis	Severe psoriasis
**VTE**			
IR (CI)<50 years	0.58 (0.57–0.59)	0.73 (0.56–0.95)	2.10 (1.32–3.33)
IR (CI)≥50 years	2.03 (2.01–2.05)	2.74 (2.45–3.06)	3.93 (3.01–5.13)

IR: incidence rate; CI: 95% confidence interval; VTE: venous thromboembolism.

### Overall and age-stratified time-dependent multivariable adjusted Poisson regression

Psoriasis was associated with a severity- and age-dependent increased risk of VTE. The overall RR was 1.35 (CI 1.21–1.49) and 2.06 (CI 1.63–2.61) for mild and severe psoriasis, respectively. The corresponding overall risks for pulmonary embolism were comparable with RRs 1.14 (CI 0.95–1.37) and 1.88 (CI 1.22–2.89). Age-stratified estimates for VTE are presented in Table 3.

**Table 3:** Adjusted age-stratified rate ratios and 95% confidence intervals of venous thromboembolism (VTE).

	Controls	Mild psoriasis	Severe psoriasis
**VTE**			
IR (CI)<50 years	1.00	1.24 (0.97–1.58)	3.14 (1.98–4.97)
IR (CI)≥50 years	1.00	1.26 (1.13–1.42)	1.74 (1.32–2.28)

RR: rate ratio; CI: 95% confidence interval.

### Sensitivity analyses: Influences of secondary VTE and surveillance bias

Exclusion of all subjects with a history of cancer or rheumatological disease did not attenuate the association between psoriasis and VTE, with RRs 1.34 (CI 1.21–1.49) and 1.99 (CI 1.56–2.53) for mild and severe psoriasis, respectively. Furthermore, censoring of participants undergoing a surgical procedure had no significant influence on the VTE risk estimates, i.e., RR 1.20 (CI 0.96–1.51) and 2.55 (CI 1.53–4.24) for mild and severe psoriasis, respectively.

With use of the less restrictive psoriasis diagnosis criterion, where patients with psoriasis were identified by their first vitamin D prescription claim or first psoriasis diagnosis, a total of 55,422 patients with mild psoriasis and 11,532 patients with severe psoriasis were included, and 22,018 patients with prevalent psoriasis were excluded. This psoriasis diagnosis definition corresponded to a 10-year psoriasis prevalence of 2.2%, and the VTE risk estimates obtained hereby were comparable to the results of the primary analyses, including the observed dose-response relationship with psoriasis severity, with overall RRs 1.44 (CI 1.34–1.56) and 1.88 (CI 1.62–2.19) for mild and severe psoriasis, respectively. Exclusion of all subjects with a history of out-patient and/or in-patient hospital contacts up to 1 year prior to study start also did not alter the results (mild psoriasis RR 1.33 [CI 1.19–1.48] and severe psoriasis 1.93 [CI 1.50–2.48]).

## Discussion

This nationwide study of VTE risk in patients with psoriasis suggested an association between psoriasis and increased risk of VTE. The present study is, to our knowledge, the first to examine the psoriasis-related risk of VTE in a large unselected cohort. We demonstrated a clear dose-response relationship between VTE risk and psoriasis severity, and the results were further corroborated by sensitivity analyses addressing the influence of secondary VTE and surveillance bias, respectively. These findings indicate that VTE should be added alongside the list of adverse atherothrombotic events, e.g., acute myocardial infarction, which has been linked to psoriasis [3]–[12].

Psoriasis is an immunoinflammatory disease characterized by T-helper (Th)1- and Th17-driven inflammation with a striking overlap of inflammatory markers and mediators with atherosclerosis [1], [26]. Indeed, these mechanisms may form the background, in part, for the increasing evidence linking psoriasis to risk of atherothrombotic cardiovascular events, e.g., acute myocardial infarction and cardiovascular mortality [3]–[12]. While it is well-established that inflammation plays a central role in the pathogenesis of atherosclerosis and atherothrombosis [26], the potential association between inflammation as determined by circulating levels of inflammatory markers (e.g., C-reactive protein) and risk of VTE is, however, unclear at present [19], [27], More than three decades ago, a small case-control study suggested that the risk of VTE and other adverse cardiovascular events was increased in psoriatic patients, but the risk of VTE was not specifically addressed in that particular study of highly selected patients [6], On the other hand, VTE has been associated with the increased risk of atherothrombotic events and was recently also linked to inflammatory bowel disease [16]–[20], In addition to increased markers of systemic inflammation, a psoriasis severity-dependent increase in platelet activation, e.g., platelet hyperaggregability and increased levels of platelet-derived microparticles and soluble P-selectin, has been demonstrated [14], [15]. Along this line, platelet activity regeneration time following aspirin ingestion may be shorter in psoriasis patients compared to controls [28], and hyperhomocysteinemia, which may be associated with atherothrombosis and VTE, has been reported in these patients, suggestive of a prothrombotic predisposition [13], [14]. Moreover, methotrexate is commonly used in severe psoriasis and though this agent may be associated with lower risk of atherothrombosis in patients with psoriasis and psoriatic arthritis, it is also associated with hyperhomocysteinemia [3], [29]. Finally, it is also interesting, that statins, which have pleiotropic effects including anti-inflammatory properties, may exert beneficial actions on psoriatic skin manifestations and may lower the risk of VTE in healthy individuals and in patients with atherosclerosis [30], [31]. In view of the considerations presented above, several mechanisms may contribute to the increased risk of VTE in patients with psoriasis, e.g., coincident risk factors, inflammation and hypercoagulability, and more studies are clearly warranted to examine individual merits of these contributions and, for example, the potential contribution of VTE to the increased cardiovascular and overall mortality observed in patients with severe psoriasis [10]–[12], [32]. In addition to the plausible pathophysiological mechanisms, the likelihood of a causal relationship between psoriasis and augmented risk of VTE as found in our study was strengthened by our study cohort design which controlled for important measured confounders, the finding of a consistent dose-response relationship between psoriasis severity and risk of VTE, and the sensitivity analyses that indicated that the elevated VTE risk was not driven by secondary VTE.

Some further strengths and limitations of the study should be discussed. The main limitation of our study is inherent to its observational nature that precludes conclusions on causality. The large number of participants, the nationwide coverage of prospectively recorded registries, and the complete follow-up strengthens the study. Specifically, the use of nationwide registries of hospitalizations and dispensed prescriptions from all pharmacies in Denmark where healthcare is readily accessible and essentially free of charge enabled us to reduce potential surveillance bias and avoid selection bias related to e.g., subject gender, age, socioeconomic status, healthcare insurance, and labor market association. This was further supported by sensitivity analyses addressing this key issue. The use of prospectively identified patients with psoriasis ensured a rational basis for allocation of exposure time and, due to the very large sample size, did not imply any significant loss of information. We were, however, unable to identify patients with psoriasis treated with topical corticosteroids alone (due to the liberal indications for these agents), and the results may not apply to these patients. Since data of in-hospital treatment are, at present, not available and as systemic antiinflammatory psoriasis treatment, i.e., methotrexate and biological agents, is generally provided directly to the patients by the respective hospital departments and are therefore not captured by the registers, we were unable to use systemic anti-inflammatory treatment as a measure of disease severity and could not address the potential impact of various systemic treatment strategies on the risk of VTE. Indeed, future studies to address the impact of various psoriasis treatment strategies on cardiovascular risk are likely to provide important insights [33]. Also, the use of hospitalizations and prescription claims to describe comorbidity, concomitant medication, and outcomes implies a possibility of underestimation of these variables since we were unable to capture data from those who did not come to medical attention. The possibility of residual confounding is always present in observational studies, and we were unable to account for some important risk factors of VTE, e.g., obesity and smoking. Importantly, we controlled for socioeconomic status that correlate with the presence of obesity and smoking. Finally, the Danish population is predominantly of Caucasian descent and extrapolation to other ethnicities should only be done with caution.

### Conclusion

This first nationwide cohort study indicates that patients with psoriasis are at increased risk of VTE. The risk was highest in young patients with severe disease. Further prospective studies are needed to confirm this association, but physicians should be aware that patients with psoriasis may be at increased risk of both venous and arterial thromboembolic events.
